# Supplementary material for: All-at-once RNA folding with 3D motif prediction framed by evolutionary information
Source: Nat Methods. 2025 Oct 3;22(10):2094–106. doi: 10.1038/s41592-025-02833-w (PMC12510878; doi:10.1038/s41592-025-02833-w)
Supplement: Supplementary file 3 — Source code to run CaCoFold-R3D. Data and results. [file 41592_2025_2833_MOESM3_ESM.gz › supplemental_material/results/CaCoFold-R3D/CaCoFold-R3D_Rfam_vshuffle/RF01574_DdR6.R2R.sto.pdf]

RF01574_DdR6

5′-U Y A A U G A U G A ● A U A A U U U A U C C C U G U C U U A G Y ○ U U U C U G U C ● U G C U G A R A A A ● ○ U G ● U R G C C U G U G G U A A G G ○ C U U U ● A C C U G A C A
